# Occurrence of virulence factors and antimicrobial resistance in *Pasteurella multocida* strains isolated from slaughter cattle in Iran

**DOI:** 10.3389/fmicb.2014.00536

**Published:** 2014-10-14

**Authors:** Faham Khamesipour, Hassan Momtaz, Morteza Azhdary Mamoreh

**Affiliations:** ^1^Young Researchers and Elite Club, Shahrekord Branch, Islamic Azad UniversityShahrekord, Iran; ^2^Department of Microbiology, Faculty of Veterinary Medicine, Shahrekord Branch, Islamic Azad UniversityShahrekord, Iran; ^3^Faculty of Veterinary Medicine, Shahrekord Branch, Islamic Azad UniversityShahrekord, Iran

**Keywords:** *Pasteurella multocida*, virulence factors, antimicrobial resistance, cattle, Iran

## Abstract

A total of 30 *Pasteurella multocida* strains isolated from 333 pneumonic and apparently health slaughter cattle were examined for capsule biosynthesis genes and 23 virulence-associated genes by polymerase chain reaction (PCR). The disc diffusion technique was used to determine antimicrobial resistance profiles among the isolates. Of the isolates, 23 belonged to capsular type A, 5 to capsular type D and two isolates were untypeable. The distribution of the capsular types in pneumonic lungs and in apparently health lungs was statistically similar. All virulence genes tested were detected among the isolates derived from pneumonic lungs; whereas isolates derived from apparently health lungs carried 16 of the 23 genes. The frequently detected genes among isolates from pneumonic lungs were *exbD, hgbA, hgbB, ompA, ompH, oma87*, and *sodC*; whereas *tadD, toxA, and pmHAS* genes occurred less frequently. Most of the adhesins and superoxide dismutases; and all of the iron acquisition and protectin proteins occurred at significantly (*p* ≤ 0.05) higher frequencies in isolates from pneumonic lungs. Isolates from apparently healthy lungs didn't carry the following genes; *hsf-1, hsf-2, tadD, toxA, nanB, nanH*, and *pmHAS*. One adhesion (*hsf-1*) and two iron acquisition (*exbD* and *tonB*) genes occurred at significantly (*p* ≤ 0.05) higher frequencies among capA isolates. All the *P. multocida* isolates were susceptible to ciprofloxacin, co-trimoxazole, doxycycline, enrofloxacin, nitrofurantoin, and tetracyclines. Different proportions of the isolates were however resistant to ampicillin, amoxicillin, erythromycin, lincomycin, penicillin, rifampin, streptomycin, and florfenicol. Our results reveal presence of virulence factors (VFs) in *P. multocida* strains isolated from symptomatic and asymptomatic bovids. A higher frequency of the factors among isolates from symptomatic study animals may suggest their role in pathogenesis of *P. multocida*-associated bovine respiratory disease (BRD). The results further reveal occurrence of antimicrobial resistance among some isolates. Control strategies for this pathogen, which could include development of an effective vaccine, are warranted so as to mitigate the social and economic consequences attributable to natural infections with this bacterium.

## Introduction

Cattle rearing is one of the important sources of income in Iran, involving both dairy and beef breeds. The sector faces a number of constraints ranging from limited feed resources to diseases. Of the diseases, those caused by infectious agents are of great importance which include bacteria and viruses affecting the respiratory system (Hemmatzadeh et al., 2001; Haji Hajikolaei and Seyfi Abad Shapouri, 2007; Sakhaee et al., 2009). The most important bacteria that play a role in pneumonia include: *Mannheimia haemolytica, Pasteurella multocida*, and *Haemophilus somnus pneumonia*, which presence of these bacteria in pneumonia lesions of slaughtered cattle around Iran have also been reported (Haji Hajikolaei et al., 2010).

Bovine respiratory disease (BRD) is a significant cause of morbidity and mortality among beef cattle in the world (Dagleish et al., 2010; Hotchkiss et al., 2010; Portis et al., 2012). Among others, *Pasteurella multocida* has been identified as a major bacterial etiologic agent for this disease (Confer, 2009; Griffin et al., 2010). It is a zoonotic Gram negative bacterium responsible for a range of infections in domestic animals causing substantial economic losses (Steen et al., 2010). The organism causes fowl cholera in domestic and wild birds, bronchopneumonia and hemorrhagic septicemia in bovids, atrophic rhinitis in porcines and snuffles in rabbits (Mannheim, 1984; Hunt et al., 2000). Most human infections with *P. multocida* result from dog and cat bites, but infections through the respiratory tract may also occur (Hubbert and Rosen, 1970).

Several host and pathogen-specific attributes do determine the outcome of infections caused by *P. multocida* (Verma et al., 2013). Of the pathogen factors important ones include the capsular and virulence-associated genes (Katsuda et al., 2013). These virulence factors (VFs) and outer membrane proteins are important for pathogenesis, functionality, protective immunity and vaccine development against *P. multocida* infections (Harper et al., 2006; Hatfaludi et al., 2010). Based on capsular antigens, *P. multocida* strains are differentiated into five serogroups i.e., type A causing fowl cholera pathogen and bovine shipping fever, type B causing hemorrhagic fever in ungulates, type D causing atrophic rhinitis in swine, type E, an African serotype, infecting cattle and buffalo; and type F also causing fowl cholera (Carter, 1955, 1961, 1967; Rimler and Rhoades, 1987). Virulence associated genes described for *P. multocida* isolates and their examples include adherence and colonization factors (*ptfA, fimA, hsf-1, hsf-2, pfhA*, and *tadD*), iron-regulated and acquisition proteins(*exbB, exbD, tonB, hgbA, hgbB*, and *Fur*), extracellular enzymes such as neuraminidase (*nanB* and *nanH*), hyaluronidase (*pmHAS*) and superoxide dismutases (*soda, sodC*, and *tbpA*), toxins (*toxA*), lipopolysaccharides (LPS), capsule and a variety of outer membrane proteins such as protectins (*ompA, omph, oma87*, and *plpB*) (Katoch et al., 2014).

Increased use of antibiotics in modern animal production has been associated with emergence of antimicrobial resistant bacteria with potential for transfer of resistance from animals to humans (Witte, 1998). As a result, antimicrobial resistance among bacterial pathogens has of recent become a big problem in both the veterinary and human medicine fields (Levy, 1998; Caprioli et al., 2000; Kehrenberg et al., 2001; White et al., 2002; Shea, 2003). The implication of the problem is increased treatment cost, prolonged illness due to treatment failure and sometimes death (Kelly et al., 2004).

The present study was conducted with the aim to detect the occurrence of VFs in *P. multocida* isolated from pneumonic and apparently health lungs of slaughter cattle in Iran. It was also to determine the occurrence of antimicrobial resistance among the isolates.

## Materials and methods

### Sample collection

A total of 333 samples, from both pneumonic (219) and apparently healthy (114) lungs, were collected randomly from slaughter cattle in an industrial abattoir in Shahrekord province during the period of September 2013 to March 2014. The abattoir receives cattle from different herds within and outside the province. For the purpose of this study pneumonic lungs referred to those lungs with gross lesions such as consolidation, fibrin deposition on the pleura, pleurisy, and/or adhesion; and apparently healthy lungs was used to describe those lungs without gross lesions. A simple random procedure was used to select pre-identified pneumonic and apparently health lungs. Random numbers were generated in Microsoft excel®. Specimens were obtained aseptically using a sterile scalpel while taking precautions to prevent surface contamination. Following collection the samples were conveyed to the microbiology laboratory in special ice-filled containers within 6 h of sampling.

### *P. multocida* screening

Isolation of *P. multocida* was done using techniques described previously by other authors (Songer and Post, 2005). Briefly, swabs were obtained from the collected samples and were plated on tryptic soy agar (Difco, Detroit, MI) containing 10 μg/ml NAD (Sigma, St. Louis, MO) and 5% bovine serum, MacConkey agar, and blood agar (5% fresh sheep blood). All plates were incubated at 37°C in air for a minimum of 48 h.

### Identification of isolates

Preliminary identification of *P. multocida* isolates was carried out according to standard biochemical tests as described earlier (Songer and Post, 2005). The isolates were gram-negative coccobacilli and were indole, catalase and oxidase-positive. But, citrate, Methyl red (MR), Vogaes–Proskauer (VP), and gelatin liquefaction negative. They don't grow on MacConkey agar and do not show hemolysis on blood agar. Confirmation of the isolates was done by polymerase chain reaction (PCR) assay with primers specific for the amplification of the KMT1 gene, adopting the methodology previously described by Townsend et al. (1998). All confirmed isolates of *P. multocida* were subsequently characterized by capsular serotyping using PCR. Primers for amplification of hyaD-hyaC and DcbF genes were used for detection of capsular type A and capsular type D, respectively (Table 1). *P. multocida* isolates which didn't yield bands on PCR when the two primers were used were classified as untyped. Following confirmation and characterization all isolates were freeze-dried and kept at −20°C.

**Table 1:** **Primers used for the detection of serogroups in strains of *P. multocida***.

**Serogroup**	**Gene**	**Primer name**	**Primer sequence (5′–3′)**	**Amplic size (bp)**	**Anneal. Temp (^°^C)**	**Reference**
All	KMT1	KMT1T7 KMT1SP6	ATCCGCTATTTACCCAGTGG GCTGTAAACGAACTCGCCAC	460	55	Townsend et al., 1998
Capsular type A	hyaD-hyaC	CAPA-F CAPA-R	CATTTATCCAAGCTCCACC GCCCGAGAGTTTCAATCC	760	55	
Capsular type D	DcbF	CAPD-F CAPD-R	TTACAAAAGAAAGACTAGGAGCCC CATCTACCCACTCAACCATATCAG	657	55	

### Detection of virulence genes

The virulence genes of *P. multocida* isolates were detected by PCR. They included adhesins (*ptfA, fimA, hsf-1, hsf-2, pfhA*, and *tadD*), toxin (*toxA*), iron acquisition (*exbB, exbD, tonB, hgbA, hgbB*, and *Fur*), sialidases (*nanB* and *nanH*), hyaluronidase (*pmHAS*), protectins (*ompA, omph, oma87*, and *plpB*) and superoxide dismutases (*soda, sodC*, and *tbpA*) (Table 2). The base sequences and the predicted sizes of the amplified products for the specific oligonucleotide primers used in detection of the genes in this study are shown in Table 3. The bacterial lysates used as templates for the PCR were prepared as follows. A loopful of bacteria from a fresh overnight culture on a tryptic soy agar plate was resuspended homogeneously in 200 μl of sterile water, and the mixture was boiled at 100°C for 5 min to release the DNA and centrifuged. A 4 μl volume of the supernatant was used as a template for each 25 μl PCR mixture. The amplified products were analyzed in 1% agarose gels by electrophoresis, and the results were recorded with a gel documentation system. All tests were repeated three times in parallel with the relevant positive (*P. multocida* strains ATCC 15742, ATCC 12945, and ATCC 12946) and negative (distilled water) controls. Discrepant results for each VF were investigated further, and samples were sequenced for gene verification.

**Table 2:** **Tested virulence-associated genes in strains of *P. multocida***.

**Gene function and gene**	**Description**
**ADHESINS**	
ptfA	Type 4 fimbriae
fimA	Fimbriae (from Pm70)
hsf-1	Autotransporter adhesion (from Pm70)
hsf-2	Autotransporter adhesion (from Pm70)
pfhA	Filamentous hemagglutinin
tadD	Putative non-specific tight adherence protein D
toxA	Dermonecrotic toxin
exbB	Accessory protein Ton-dependent transport of iron compounds
exbD	Accessory protein Ton-dependent transport of iron compound
tonB	Iron transporters, transport ferric-siderophore complexes
hgbA	A hemoglobin-binding protein
hgbB	B hemoglobin-iron uptake
Fur	Ferric uptake regulation protein
**SIALIDASES nanB**	
nanB	Outer membrane-associated proteins, an autotransporter protein
nanH	Outer membrane-associated proteins, small sialidases
**HYALURONIDASE**	
pmHAS	Hyaluronan synthase
**SUPEROXIDE DISMUTASE**	
sodA	Superoxide dismutase
sodC	Superoxide dismutase
tbpA	Superoxide dismutase
**PROTECTINS**	
ompA	Outer membrane protein A
ompH	Outer membrane protein H
oma87	Outer membrane protein 87
plpB	Lipoprotein B

**Table 3:** **Primers used for the detection of virulence-associated genes in strains of *P. multocida***.

**Gene function and gene**	**Primer sequence (5′–3′)**	**Amplicon size (bp)**	**Annealing temp (^°^C)**	**References**
**ADHESINS**				
ptfA	TGTGGAATTCAGCATTTTAGTGTGTC TCATGAATTCTTATGCGCAAAATCCT GCTGG	488	55	Townsend et al., 1998
fimA	CCATCGGATCTAAACGACCTA AGTATTAGTTCCTGCGGGTG	866	55	
hsf-1	TTGAGTCGGCTGTAGAGTTCG ACTCTTTAGCAGTGGGGACAACCTC	654	54	
hsf-2	ACCGCAACCATGCTCTTAC TGACTGACATCGGCGGTAC	433	54	
pfhA	TTCAGAGGGATCAATCTTCG AACTCCAGT TGGTTTGTCG	286	55	
tadD	TCTACCCATTCTCAGCAAGGC ATCATTTCGGGCATTCACC	416	55	
**TOXINS**				
toxA	CTTAGATGAGCGACAAGG GAATGCCACACCTCTATAG	864	55	Townsend et al., 1998
**SUPEROXIDE DISMUTASE**				
sodA	TACCAGAATTAGGCTACGC GAAACGGGTTGCTGCCGCT	361	55	Ewers et al., 2006
tbpA	TTGGTTGGAAACGGTAAAGC TAACGTGTACGGAAAAGCCC	728	54	
sodC	AGTTAGTAGCGGGGTTGGCA TGGTGCTGGGTGATCATCATG	235	55	Lainson et al., 1996
**SIALIDASES nanB**				
nanB	CATTGCACCTAACACCTCT GGACACTGATTGCCCTGAA	555	55	Townsend et al., 1998
nanH	GTGGGAACGGGAATTGTGA ACATGCCAAGTTTGCCCTA	287	55	
**PROTECTINS**				
ompA	CGCATAGCACTCAAGTTTCTCC CATAAACAGATTGACCGAAACG	201	55	Townsend et al., 1998
ompH	CGCGTATGAAGGTTTAGGT TTTAGATTGTGCGTAGTCAAC	438	55	
oma87	GGCAGCGAGCAACAGATAACG TGTTCGTCAAATGTCGGGTGA	838	55	
plpB	TTTGGTGGTGCGTATGTCTTCT AGTCACTTTAGATTGTGCGTAG	282	55	
**HYALURONIDASE**				
pmHAS	TCAATGTTTGCGATAGTCCGTTAG TGGCGAATGATCGGTGATAGA	430	54	Townsend et al., 1998
**IRON ACQUISITION**				
exbB	TTGGCTTGTGATTGAACGC TGCAGGAATGGCGACTAA A	283	55	Townsend et al., 1998
exbD	CGTTCTGATTACAGCCTCTT AACGAAATCTTGGAAACTGG	247	55	
tonB	CGACGGTGAAACCTGAGCCA CCGAGCGATAAGCATTGACT	261	55	
hgbA	TCAACGGCAGATAATCAGGG GCGGGAATGCTGAAGATAAG	267	55	
Fur	GTTTACCGTGTATTAGACCA CATTACTACATTTGCCATAC	244	55	
hgbB	ACCGCGTTGGAATTATGATTG CATTGAGTACGGCTTGACAT	788	55	Ewers et al., 2006

### Antimicrobial resistance test

Antimicrobial resistance profiles of the isolates to 20 antimicrobial agents were determined by the disc diffusion method on Muller Hinton agar with 5% blood (Carter and Subronto, 1978). The plates were inoculated with a cotton swab dipped into a 0.5 McFarland standard suspension of each isolates, according to the procedures outline in NCCLS (NCCLS, 2008). Then, the plates were incubated at 37°C for 24 h. The inhibition zones around each disc were measured and interpretation of results made according to the guidelines provided by manufacturers (Pattan-Teb, Tehran, Iran) and those provided by NCCLS (2008). The results were interpreted as resistant (R), intermediate (I), and susceptible (S).

### Statistical data analysis

Data analysis was performed in SPSS software version 12.0 (SPSS Inc., Chicago, IL). Descriptive statistics were computed to determine the proportions of the different VFs among the isolates; and proportions of isolates resistant to different antimicrobial agents. Chi square test adopted for determination of statistical significance of differences between the proportions.

## Results

### Prevalence of *P. multocida* in collected samples

The prevalence of *P. multocida* in collected lung samples is indicated in Table 4. Overall 9.0% (30/333) of the sampled cattle were infected with the organism. The frequency of infection with the organism was higher in pneumonic lungs than in apparently health lungs and the difference was statistically significant at *p* ≤ 0.05.

**Table 4:** **Prevalence of *P. multocida* in collected cattle lung samples**.

**Lung samples**	**Number of samples**	**Number of positive samples**
Pneumonic lungs	219	25 (11.4%)
Healthy lungs	114	5 (4.4%)
Total	333	30 (9.0%)

### Distribution of VFs according to capsular serotypes

Two capsular types (A and D) were detected among 28 of the 30 isolates obtained as seen in Tables 5, 6. The majority (76.7%) of the isolates were of capsular type A. The distribution of the capsular types in pneumonic lungs and in apparently health lungs (Table 5) didn't show any statistically significant difference. The distribution of capsular serotypes for each individual isolate is displayed in Table 6.

**Table 5:** **Distribution of capsular serotypes among the isolates**.

**Capsular**	**Overall**	**Pneumonic lung**	**Apparently healthy**
**types**	**prevalence**	**isolates**	**lung isolates**
	**(*n* = 30)**	**(*n* = 25)**	**(*n* = 5)**
Type A	23 (76.7%)	18 (72.0%)	5 (100.0%)
Type D	5 (16.7%)	5 (20.0%)	–
Untyped	2 (6.7%)	2 (8.0%)	–

**Table 6:** **Capsular types and virulence genes detected among *P. multocida* isolates obtained from cattle lungs in Iran**.

**Strain ID**	**Capsule type**	**Virulence genes**
1	Type A	*ptfA, fimA, hsf-1, tonB, hgbA, hgbB, Fur, nanB, nanH, pmHAS, ompA, oomph, plpB, soda, sodC*
2	Type A	*ptfA, fimA, hsf-1, exbD, tonB, hgbA, hgbB, Fur, nanB, nanH, pmHAS, ompA, oomph, oma87, plpB, soda, sodC*
3	Type A	*ptfA, fimA, hsf-1, tonB, hgbA, hgbB, Fur, nanB, nanH, pmHAS, ompA, oomph, oma87, plpB, soda, sodC, tbpA*
4	Type D	*ptfA, hsf-2, pfhA, exbB, exbD, hgbA, hgbB, Fur, nanB, nanH, ompA, oomph, oma87, plpB, sodC, tbpA*
5	Type D	*ptfA, fimA, hsf-2, pfhA, tadD, exbB, hgbA, hgbB, Fur, nanB, nanH, ompA, oomph, oma87, plpB, sodC*
6	Untyped	*pfhA, Fur, nanB, nanH, ompA, oomph, oma87, plpB, tbpA*
7	Type A	*ptfA, fimA, hsf-1, hsf-2, toxA, exbD, tonB, hgbA, hgbB, Fur, nanB, nanH, pmHAS, ompA, oomph, oma87, plpB, sodA, sodC*
8	Type A	*ptfA, fimA, hsf-1, hsf-2, pfhA, exbB, exbD, tonB, hgbA, hgbB, Fur, nanB, nanH, pmHAS, ompA, oomph, oma87, plpB, soda, sodC, tbpA*
9	Type A	*ptfA, fimA, hsf-1, hsf-2, pfhA, exbB, exbD, tonB, hgbA, hgbB, Fur, nanB, pmHAS, plpB, ompA, oomph, oma87, soda, sodC, tbpA*
10	Type A	*ptfA, fimA, exbB, exbD, tonB, hgbA, hgbB, Fur, nanB, pmHAS, ompA, oma87, plpB, soda, sodC, tbpA*
11	Type D	*ptfA, fimA, hsf-2, pfhA, exbB, exbD, hgbA, hgbB, Fur, nanB, nanH, pmHAS, ompA, oma87, plpB*
12	Type A	*ptfA, fimA, hsf-2, exbB, exbD, tonB, hgbA, hgbB, pmHAS, oma87, plpB, soda, sodC*
13	Type A	*ptfA, fimA, hsf-1, hsf-2, pfhA, tadD, exbB, exbD, tonB, hgbA, hgbB, Fur, nanB, ompA, oomph, oma87, plpB, soda, sodC, tbpA*
14	Type A	*ptfA, fimA, hsf-1, hsf-2, pfhA, tadD, exbB, exbD, tonB, hgbA, hgbB, Fur, nanB, ompA, oomph, oma87, plpB, sodA, sodC, tbpA*
15	Type A	*ptfA, fimA, hsf-1, hsf-2, exbB, exbD, tonB, hgbA, hgbB, Fur, nanB, nanH, ompA, oomph, oma87, plpB, soda, sodC, tbpA*
16	Type D	*ptfA, fimA, hsf-2, tadD, exbB, exbD, hgbA, hgbB, Fur, nanB, nanH, pmHAS, ompA, oomph, oma87, plpB, sodA*
17	Type A	*ptfA, fimA, hsf-2, tadD, exbB, exbD, tonB, hgbB, Fur, nanB, nanH, ompA, oomph, oma87, plpB, soda, sodC, tbpA*
18	Type A	*ptfA, fimA, hsf-1, pfhA, toxA, exbB, exbD, tonB, hgbB, Fur, nanB, nanH, ompA, oomph, oma87, plpB, soda, sodC, tbpA*
19	Type A	*ptfA, fimA, hsf-1, hsf-2, pfhA, tadD, exbB, exbD, tonB, hgbB, Fur, nanB, nanH, ompA, oomph, oma87, plpB, soda, sodC, tbpA*
20	Type A	*ptfA, fimA, hsf-1, hsf-2, toxA, exbB, exbD, tonB, hgbA, hgbB, Fur, nanB, nanH, ompA, oomph, oma87, soda, sodC, tbpA*
21	Untyped	*fimA, exbB, hgbA, nanH, ompA, oomph, plpB*
22	Type A	*ptfA, fimA, hsf-1, hsf-2, pfhA, exbB, exbD, tonB, hgbA, hgbB, Fur, nanB, nanH, ompA, oomph, oma87, plpB, soda, sodC, tbpA*
23	Type A	*ptfA, fimA, hsf-2, exbB, exbD, tonB, hgbA, hgbB, Fur, nanB, nanH, ompA, oomph, oma87, plpB, soda, sodC, tbpA*
24	Type A	*ptfA, fimA, hsf-2, pfhA, tadD, exbB, exbD, tonB, hgbA, hgbB, nanB, nanH, ompA, oomph, oma87, plpB, soda, sodC, tbpA*
25	Type A	*ptfA, fimA, hsf-1, hsf-2, pfhA, tadD, exbB, tonB, hgbA, hgbB, Fur, nanB, nanH, oomph, oma87, plpB, soda, sodC, tbpA*
26	Type D	*ptfA, hsf-2, pfhA, exbB, hgbA, hgbB, nanB, Fur, ompA, oma87, plpB, soda, sodC*
27	Type A	*hsf-1, hsf-2, pfhA, tadD, exbB, exbD, tonB, hgbA, hgbB, Fur, nanB, nanH, ompA, oomph, oma87, soda, sodC, tbpA*
28	Type A	*fimA, hsf-1, hsf-2, pfhA, tadD, exbB, exbD, tonB, hgbA, hgbB, Fur, nanH, oomph, oma87, soda, sodC, tbpA*
29	Type A	*hsf-1, hsf-2, pfhA, tadD, exbB, exbD, tonB, hgbA, hgbB, ompA, nanH, oomph, soda, sodC, tbpA*
30	Type A	*hsf-1, hsf-2, pfhA, tadD, exbB, exbD, tonB, hgbA, hgbB, nanH, ompA, oomph, oma87, soda, sodC*

### Distribution of VFs according to associated VF genes

All isolates from pneumonic lungs harbored at least one virulence gene as displayed in Table 7. Table 8 shows the distribution of virulence genes by capsular serotypes. The detected virulence genes for each isolate obtained in this study is presented in Table 6. Most of the adhesins and superoxide dismutases; and all of the iron acquisition and protectin proteins occurred at significantly (*p* ≤ 0.05) higher frequencies in isolates from pneumonic lungs. One adhesion (*hsf-1*) and two iron acquisition (*exbD* and *tonB*) genes occurred at significantly (*p* ≤ 0.05) higher frequencies among capA isolates.

**Table 7:** **Distribution of VFs according to associated VF genes**.

**Virulence genes**	**Overall prevalence (*n* = 30)**	**Pneumonic lung isolates (*n* = 25)**	**Apparently healthy lung isolates (*n* = 5)**
**ADHESINS**			
*ptfA*	24 (80.0%)	23 (92.0%)	1 (20.0%)
*fimA*	24 (80.0%)	23 (92.0%)	1 (20.0%)
*hsf-1*	18 (60.0%)	18 (72.0%)	–
*hsf-2*	23 (76.7%)	23 (92.0%)	–
*pfhA*	18 (60.0%)	15 (60.0%)	3 (60.0%)
*tadD*	12 (40.0%)	12 (48.0%)	–
**TOXINS**			
*toxA*	3 (10.0%)	3 (12.0%)	–
**IRON ACQUISITION**			
*exbB*	25 (83.3%)	24 (96.0%)	1 (20.0%)
*exbD*	26 (86.7%)	25 (100.0%)	1 (20.0%)
*tonB*	25 (83.3%)	24 (96.0%)	1 (20.0%)
*hgbA*	26 (86.7%)	25 (100.0%)	1 (20.0%)
*hgbB*	28 (93.3%)	25 (100.0%)	3 (60.0%)
*Fur*	25 (83.3%)	24 (96.0%)	1 (20.0%)
**SIALIDASES nanB**			
*nanB*	25 (83.3%)	25 (100.0%)	–
*nanH*	24 (80.0%)	24 (96.0%)	–
**HYALURONIDASE**			
*pmHAS*	10 (33.3%)	10 (40.0%)	–
**PROTECTINS**			
*ompA*	27 (90.0%)	25 (100.0%)	2 (40.0%)
*ompH*	26 (86.7%)	25 (100.0%)	1 (20.0%)
*oma87*	27 (90.0%)	25 (100.0%)	2 (40.0%)
*plpB*	25 (83.3%)	24 (96.0%)	1 (20.0%)
**SUPEROXIDE DISMUTASE**			
*sodA*	25 (83.3%)	24 (96.0%)	1 (20.0%)
*sodC*	26 (86.7%)	25 (100.0%)	1 (20.0%)
*tbpA*	20 (66.7%)	18 (72.0%)	2 (40.0%)

**Table 8:** **Distribution of VFs according to capsule serotypes among 30 bovine isolates of *P. multocida***.

**Virulence genes**	**Overall (*n* = 30)**	**capA (*n* = 23)**	**capD (*n* = 5)**	**Untyped (*n* = 2)**
**ADHESINS**				
*ptfA*	24 (80.0%)	19 (82.6%)	5 (100.0%)	–
*fimA*	24 (80.0%)	20 (87.0%)	3 (60.0%)	1 (50.0%)
*hsf-1*	18 (60.0%)	18 (78.3%)	–	–
*hsf-2*	23 (76.7%)	18 (78.3%)	5 (100.0%)	–
*pfhA*	18 (60.0%)	13 (56.5%)	4 (80.0%)	1 (50.0%)
*tadD*	12 (40.0%)	10 (43.5%)	2 (40.0%)	–
**TOXINS**				
*toxA*	3 (10.0%)	3 (13.0%)	–	–
**IRON ACQUISITION**				
*exbB*	25 (83.3%)	19 (82.6%)	5 (100.0%)	1 (50.0%)
*exbD*	26 (86.7%)	23 (100.0%)	3 (60.0%)	–
*tonB*	25 (83.3%)	23 (100.0%)	–	2 (100.0%)
*hgbA*	26 (86.7%)	20 (87.0%)	5 (100.0%)	1 (50.0%)
*hgbB*	28 (93.3%)	23 (100.0%)	5 (100.0%)	–
*Fur*	25 (83.3%)	19 (82.6%)	5 (100.0%)	1 (50.0%)
**SIALIDASES nanB**				
*nanB*	25 (83.3%)	19 (82.6%)	5 (100.0%)	1 (50.0%)
*nanH*	24 (80.0%)	18 (78.3%)	4 (80.0%)	2 (100.0%)
**HYALURONIDASE**				
*pmHAS*	10 (33.3%)	8 (34.8%)	2 (40.0%)	–
**PROTECTINS**				
*ompA*	27 (90.0%)	20 (87.0%)	5 (100.0%)	2 (100.0%)
*ompH*	26 (86.7%)	21 (91.3%)	3 (60.0%)	2 (100.0%)
*oma87*	27 (90.0%)	21 (91.3%)	5 (100.0%)	1 (50.0%)
*plpB*	25 (83.3%)	18 (78.3%)	5 (100.0%)	2 (100.0%)
**SUPEROXIDE DISMUTASE**				
*sodA*	25 (83.3%)	23 (100.0%)	2 (40.0%)	–
*sodC*	26 (86.7%)	23 (100.0%)	3 (60.0%)	–
*tbpA*	20 (66.7%)	18 (78.3%)	1 (20.0%)	1 (50.0%)

### Antimicrobial resistance among the isolates

Antimicrobial resistance profiles of *P. multocida* isolates obtained in this study are displayed in Table 9. All the isolates were susceptible to ciprofloxacin, co-trimoxazole, doxycycline, enrofloxacin, nitrofurantoin, and tetracyclines. Resistance to ampicillin, lincomycin, penicillin, rifampin, streptomycin, amoxicillin, erythromycin, and florfenicol was observed at different frequencies.

**Table 9:** **Antimicrobial resistance profiles of *P. multocida* isolates against 20 antimicrobial agents**.

**Antimicrobial agent**	**Resistant isolates**	**Intermediate resistant isolates**	**Susceptible isolates**
Ampicillin	10 (33.3%)	11 (36.7%)	9 (30.0%)
Amikacin	0 (0.0%)	1 (3.3%)	29 (96.7%)
Cloramphenicol	0 (0.0%)	0 (0.0%)	29 (96.7%)
Carbenicillin	0 (0.0%)	1 (3.3%)	29 (96.7%)
Ciprofloxacin	0 (0.0%)	0 (0.0%)	30 (100.0%)
Co-trimoxazole	0 (0.0%)	0 (0.0%)	30 (100.0%)
Doxycycline	0 (0.0%)	0 (0.0%)	30 (100.0%)
Enrofloxacin	0 (0.0%)	0 (0.0%)	30 (100.0%)
Gentamicin	0 (0.0%)	1 (3.3%)	29 (96.7%)
Lincomycin	13 (43.3%)	8 (26.7%)	8 (26.7%)
Nitrofurantoin	0 (0.0%)	0 (0.0%)	30 (100.0%)
Oxytetracycline	0 (0.0%)	0 (0.0%)	30 (100.0%)
Penicillin	12 (40.0%)	9 (30.0%)	9 (30.0%)
Rifampin	6 (20.0%)	6 (20.0%)	18 (60.0%)
Streptomycin	5 (16.7%)	0 (0.0%)	25 (83.3%)
Tetracycline	0 (0.0%)	0 (0.0%)	30 (100.0%)
Amoxicillin	3 (10.0%)	3 (10.0%)	24 (80.0%)
Erythromycin	10 (33.3%)	10 (33.3%)	10 (33.3%)
Kanamycin	0 (0.0%)	4 (13.3%)	26 (86.7%)
Florfenicol	5 (16.7%)	6 (20.0%)	19 (63.3%)

## Discussion

VFs play a key role in disease production by bacterial pathogens (Nanduri et al., 2009). Among others, their functions include competence, adherence, synthesis, and export of capsules; and evasion of host immune responses (Nanduri et al., 2009). In the present study the factors have been detected in *P. multocida* isolated from the lungs of slaughter cattle. The higher frequency of the factors among isolates from pneumonic lungs suggests the role of these factors in disease occurrence. It was pointed out that virulence gene occurrence in *P. multocida* has a strong positive association with the outcome of infection with the organism in cattle (Katsuda et al., 2013). On the other hand occurrence of the factors in apparently healthy lungs could possibly indicate early infection or contained infection which couldn't lead to disease. It was previously reported that this facultative anaerobic bacterium is commonly found in clinically healthy calves (Lainson et al., 2013).

In this study capsular types A and D were detected using PCR among the obtained *P. multocida* isolates. A small proportion (6.7%; 2/30) of *P. multocida* strains were untypeable, a similar observation to what was reported by Arumugam et al. (2011). Capsular type A was predominant among the strains accounting for 76.6%. Our observation is similar to a finding by Katsuda et al. (2013) who also detected capsular types A and D among cattle derived *P. multocida* isolates; with type A occurring at higher frequency. A higher frequency of capsular type A among cattle derived *P. multocida* isolates has also been reported in a study conducted earlier by Davies et al. (2004) who found that 99.3% of bovine *P. multocida* strains (*n* = 153) were of this capsular type. *P. multocida* isolates of serotype A are common in bovids occurring as normal flora in the nasopharynx; or as causes of disease including BRD and hemorraghic septicemia (Ewers et al., 2004; Dabo et al., 2007). The capsular type A is also most frequently described for rabbits (Ewers et al., 2006) and pigs (García et al., 2011).

Of the protectins; *OmpA* and *oma87* were the most frequently detected genes particularly in the isolates from pneumonic lungs. Slightly higher frequencies of the two genes were noted for isolates of the capA serogroup than those belonging to the capD serogroup. The *OmpA* gene has a significant role in stabilizing the cell envelope structure by providing physical linkage between the outer membrane and peptidoglycan (Katoch et al., 2014). It mediates *P. multocida* host cells interaction through heparin and/or fibronectin binding and thus acts as an important invasive molecule which could determine the outcome of infection with the organism (Katoch et al., 2014).

The type 4 fimbria (ptfA gene) was described in 92.0% of the isolates tested in the current study. The gene plays a key role of fixing bacterial pathogens on the surface of the epithelial cells of hosts, a phenomenon which is more common in rabbits (Ewers et al., 2006). Consequently a study conducted on rabbits described a high prevalence of the ptfA gene (93.4%; 43/46).

Presence of adhesins on the bacterial surface is usually linked to virulence as these proteins are known to play a crucial role in facilitating host invasion and colonization (Kline et al., 2009). Studies by Ewers et al. (2000) and Tang et al. (2009) have demonstrated that, of the adhesins; fimA, hsf-2, and ptfA are of frequent occurrence among pathogenic isolates of *P. multocida*. In the current study the three adhesins were demonstrated at higher frequencies than others in both capA and capD serogroup isolates. On the other hand, gene tadD was the least frequently detected adhesin among *P. multocida*, occurring only in 48.0% of the isolates (*n* = 30). In these organisms the gene is known to be a putative non-specific tight adherence protein D (May et al., 2001). A more or less similar low frequency (43.3%; 100/233) of tadD was described in a field study involving pigs (Tang et al., 2009). A work on rabbits, however, observed a higher frequency (91.3%; 42/46) of this gene among *P. multocida* strains.

It is noteworthy that the dermonecrotoxin encoding toxA was the least frequently detected gene among the isolates; demonstrated only in those of capsular type A obtained from pneumonic lungs. Some other researchers indicated that this particular gene is more frequently expressed by strains of serogroup D and is responsible for the clinical symptoms associated with atrophic rhinitis in porcines (Harper et al., 2006; Ferreira et al., 2012). The observation in the current study could be attributed to the small sample size of capsular type D isolates. In a study conducted earlier the gene was detected in *P. multocida* isolates from avians, swine, shoats and cattle; but was only associated with disease in pigs (Ewers et al., 2006). Pullinger et al. (2004) points out that the toxA gene is not inserted into the bacterial chromosome but in a lysogenic bacteriophage that infects the agent.

The *tbpA* encoding gene is known to be of common occurrence among ruminant *P. multocida* strains (Ewers et al., 2006; Atashpaz et al., 2009). Its prevalence was however relatively low when compared to other superoxide dismutases (sodA and sodC) tested in this study. Ferreira et al. (2012) found a low frequency (8.6%; 4/46) of this gene in a study conducted on rabbits. Variable frequencies of the genes encoding proteins with different functions, such as adhesins (fimA, hsf-1, hsf-2, and pfhA), iron acquisition (exbB, exbD, tonB, hgbA, hgbB, and Fur), sialidases (nanB and nanH), hyaluronidase (pmHAS), and protectins (oomph and plpB) were found in the isolates. This finding is similar to what was reported in previous works which involved ruminants, porcine, poultry, and rabbits (Ewers et al., 2006; Tang et al., 2009; Ferreira et al., 2012).

Infections with *P. multocida* are commonly managed by broad-spectrum antimicrobials (Kehrenberg et al., 2001; Lion et al., 2006; Brogden et al., 2007). Studies have however reported occurrence of resistance to a large number of antimicrobial agents among *P. multocida* isolates (Hunt et al., 2001; Davies et al., 2004; Arashima and Kumasaka, 2005). In the current study all the *P. multocida* isolates were susceptible to ciprofloxacin, co-trimoxazole, doxycycline, enrofloxacin, nitrofurantoin, and tetracyclines. Similar observations for ciprofloxacin and co-trimoxazole (Mohamed et al., 2012); and for enrofloxacin, tetracycline, and doxycycline (Ferreira et al., 2012) have also been made earlier. These antibiotics can therefore be used for prevention and treatment of bovine *P. multocida* infections in the study area. Unlike other authors who reported poor (Gutiérrez Martin and Rodríguez Ferri, 1993; Yoshimura et al., 2001) and moderate (Mohamed et al., 2012) activity of aminoglycoside antibiotics against *P. multocida*, in the present study kanamycin, gentamicin, amikacin, and streptomycin exhibited high activity against the tested isolates. The frequencies of resistant isolates to other antibiotics varied greatly as reported by other researchers (Salmon et al., 1995; Kehrenberg et al., 2001; Yoshimura et al., 2001; Welsh et al., 2004).

The major limitation in the discussion of the findings of the current study was large differences in the sample sizes of comparison groups as seen for isolates between isolates from pneumonic lungs and those from apparently health lungs; and isolates of different capsular types. This made it difficult to infer on the observed variations as they could be attributed to chance.

In summary, our results reveal presence of VFs in *P. multocida* strains isolated from the lungs of symptomatic and asymptomatic slaughter cattle. Frequent detection of the factors among isolates from symptomatic study animals may suggest their role in pathogenesis of BRD caused by these organisms. Occurrence of antimicrobial resistance among some isolates is of great concern. Control strategies for this pathogen, which could include development of an effective vaccine, are warranted so as to mitigate the social and economic consequences attributable to natural infections with this bacterium. Further, the use of antimicrobial agents in modern livestock farming need to be controlled so as to minimize the emergence and eventually spread of resistance not only in target microbes but also in other important zoonotic pathogens.

### Conflict of interest statement

The authors declare that the research was conducted in the absence of any commercial or financial relationships that could be construed as a potential conflict of interest.
